# Fuzzy Boundaries: Color and Gene Flow Patterns among Parapatric Lineages of the Western Shovel-Nosed Snake and Taxonomic Implication

**DOI:** 10.1371/journal.pone.0097494

**Published:** 2014-05-21

**Authors:** Dustin A. Wood, Robert N. Fisher, Amy G. Vandergast

**Affiliations:** U.S. Geological Survey, Western Ecological Research Center, San Diego Field Station, San Diego, California, United States of America; University of Innsbruck, Austria

## Abstract

Accurate delineation of lineage diversity is increasingly important, as species distributions are becoming more reduced and threatened. During the last century, the subspecies category was often used to denote phenotypic variation within a species range and to provide a framework for understanding lineage differentiation, often considered incipient speciation. While this category has largely fallen into disuse, previously recognized subspecies often serve as important units for conservation policy and management when other information is lacking. In this study, we evaluated phenotypic subspecies hypotheses within shovel-nosed snakes on the basis of genetic data and considered how evolutionary processes such as gene flow influenced possible incongruence between phenotypic and genetic patterns. We used both traditional phylogenetic and Bayesian clustering analyses to infer range-wide genetic structure and spatially explicit analyses to detect possible boundary locations of lineage contact. Multilocus analyses supported three historically isolated groups with low to moderate levels of contemporary gene exchange. Genetic data did not support phenotypic subspecies as exclusive groups, and we detected patterns of discordance in areas where three subspecies are presumed to be in contact. Based on genetic and phenotypic evidence, we suggested that species-level diversity is underestimated in this group and we proposed that two species be recognized, *Chionactis occipitalis* and *C. annulata*. In addition, we recommend retention of two subspecific designations within *C. annulata* (*C. a. annulata* and *C. a. klauberi*) that reflect regional shifts in both genetic and phenotypic variation within the species. Our results highlight the difficultly in validating taxonomic boundaries within lineages that are evolving under a time-dependent, continuous process.

## Introduction

Although scientific debates over species concepts persist, the notion that species exist as separately evolving lineages, presumably under various stages of speciation, is central to discussions related to intraspecific diversity. While most contemporary species concepts agree conceptually that species are separately evolving “metapopulation lineages” (i.e., the general lineage concept of a species; de Queiroz [1]) the challenge lies in determining where a species should be recognized within this continuum. Intraspecific diversity garnered early interest in the field of evolutionary biology, as Darwin [2] makes clear: “those forms which possess in some considerable degree the character of species, but which are so closely similar to some other forms, or are so closely linked to them by intermediate gradations, that naturalists do not like to rank them as distinct species, are in several respects the most important for us.” This sentiment extended through the 20^th^ century, where researchers often used the subspecific rank to fulfill two roles: (1) to denote the phenotypic variation within a species range, and (2) to provide a framework for understanding the heirarchical levels of lineage differentiation, often considered incipient speciation [3]–. During the late 20^th^ century this either-or approach to subspecies delimitation unleashed confusion on the concept of species and sparked critisism of the subspecies rank. As a result, the subspecies rank has largely fallen into disuse. However, within the field of conservation biology, historically described subspecies often serve as important units for conservation policy and management when other information is lacking [8]–[11]. Therefore, accurate delineation of lineage diversity and population genetic structure remains an important task, as species distributions are becoming ever more reduced and threatened [12]–[15].

Criticism of the subspecies rank itself has helped to bring modern statistical and genetic techniques to bear on the issue of whether recognized subspecies form natural groups. Phylogenetic methods and molecular characters are often used to identify genetic lineages and evaluate subspecies, often through the use of single locus markers (e.g. mitochondrial DNA). While numerous studies have demonstrated that phenotypic subspecies often fail to correspond to monophyletic genetic lineages, the generality of this pattern across taxa remains controversial [9], [11], [16]–[21]. Furthermore, several authors have suggested that the presence or absence of monophyly is too stringent of a criterion [22]–[24], because introgression and incomplete lineage sorting between diverging populations can lead to incongruent gene trees at the species-subspecies boundary [11], [25]. Therefore, using criteria that only emphasize historical isolation based on gene trees from single, neutrally evolving loci ignores other factors contributing to phenotypic differences that may be important for conservation. For example, local adaptation may contribute to genetic divergence, but the divergence may be so recent that insufficient time has passed to achieve reciprocally monophyletic gene trees [25], [26].

Multi-locus genetic surveys that examine larger portions of the genome have garnered broad support as a preferred strategy for studying lineage divergence and genetic differentiation [24], [27], [28]. Clustering-based approaches that define genetic clusters using multi-locus datasets can be used to evaluate units at the species-subspecies boundary [15], [24]. These methods incorporate a Bayesian framework to probabilistically cluster individuals into the most likely number of groups given the data, which can then be evaluated against how well they correspond to phenotypic subspecies circumscriptions.

**Figure 1:**
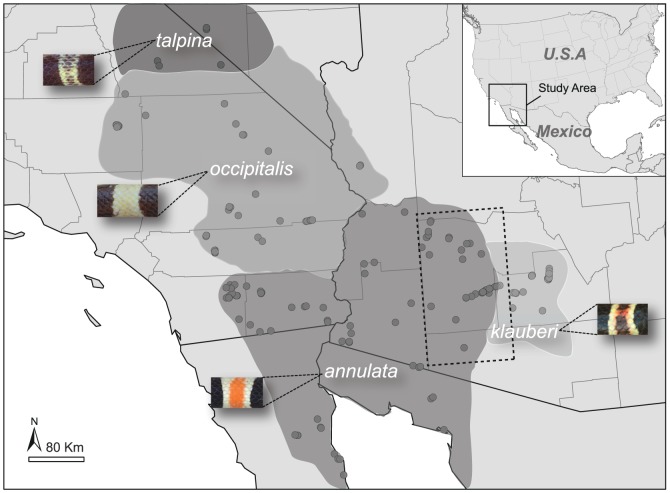
Subspecies distribution and dorsal color patterns of the western shovel-nosed snake (*Chionactis occipitalis*) along with genetic sampling locations (black dots). The presumed range of the morphological intergrade zone at the interface of *C. o. annulata* and *C. o. klauberi* is depicted by the dashed line.

In this study, we investigate the species-subspecies boundaries of Western shovel-nosed snake (*Chionactis occipitalis*) and evaluate whether the phenotypic subspecies correspond to genetic groups on the basis of multiple selectively neutral loci. *Chionactis occipitalis* is a small colubrid snake occupying the arid valley floors and bajadas of the Mojave and Sonoran deserts in southwestern North America. Four subspecies have been recognized on the basis of variation in banding pattern and coloration throughout the distribution [29]–[31] and are recognized as follows: *C. o. occipitalis* (Mojave shovel-nosed snake), *C. o. annulata* (Colorado shovel-nosed snake), *C. o. talpina* (Nevada shovel-nosed snake), and *C. o. klauberi* (Tucson shovel-nosed snake). *Chionactis occipitalis klauberi* is of particular conservation interest. This subspecies occurs along the eastern edge of the species range in south central Arizona and has experienced declines in population size and range over the past several decades due to alteration and loss of habitat through agricultural and urban development. These factors prompted a petition to list the subspecies as threatened or endangered under the U.S. Endangered Species Act [32]. At present, *C. o. klauberi* has been elevated to “candidate status” (Priority 3) under the ESA [33]–[35]. Even so, existing taxonomic uncertainty within *C. occipitalis* may hinder further management actions [35]. For example, *C. o. klauberi* was described despite the assumption that it formed a broad zone of intergradation with *C. o. annulata* across Arizona (Fig.1; [30]). Specimens taken from within the intergrade zone are often difficult to unambiguously assign to either subspecies because (i) they contain phenotypic characters consistent with *C. o. klauberi* but are found well outside the presumed geographic distribution, or (ii) specimens display intermediate morphological characteristics between the two subspecies. As a result, various authors have treated the range of *C. o. klauberi* differently [29], [36]. Therefore, one problem in determining subspecies validity lies in the fact that the distribution of *C. o. klauberi* was never clearly delineated.

Previous research by Wood *et al.* [37] investigated the genetic structuring of *C. occipitalis* using mitochondrial DNA (mtDNA) sequence data from 81 snakes and variation in 14 morphological characters (excluding color pattern) from 1543 snakes from across the range of the species. Sequence data revealed a deep phylogenetic split within the species with substantial genetic structuring within the two main clades. Most relevant to this study, mtDNA haplotypes of individuals identified as *C. o. klauberi* were intermixed with haplotypes belonging to *C. o. annulata*. Broad morphological overlap was detected between the four subspecies, and only minor geographic structure was evident for *C. o. klauberi*. Taken together, Wood *et al.* [37] suggested that *C. o. klauberi* may represent a “morphological endpoint of clinal variation without concordant phylogenetic distinction”, but encouraged further hypothesis testing with nuclear loci to evaluate the mtDNA gene tree patterns and possible ecological/adaptive differences that may exist despite non-exclusive mtDNA genetic variation.

Here, we build on this research using 11 nuclear microsatellite loci and additional mtDNA sequence data to further evaluate the genetic groups within *C. occipitalis*, placing emphasis on evaluating the genetic distinctiveness between *klauberi* and the neighboring *annulata* phenotypes across the Colorado and Sonoran deserts. Our objectives addressed three gaps in the current knowledge. First, we tested whether the nuclear genome shows patterns of structure that are consistent with the ranges of the four recognized phenotypic subspecies. Second, we tested for intergradation between *annulata* and *klauberi*, and identified the geographic location of a contact zone. Third, we quantified the extent to which the nuclear genetic clusters within *C. occipitalis* are isolated. Finally, we provide information from multiple genetic and phenotypic datasets to suggest taxonomic recommendations consistent with lineage evolution within this group.

## Materials and Methods

### Sampling and DNA extraction

We obtained 269 tissue samples of *Chionactis occipitalis* from localities throughout California, Arizona, Nevada, Baja California and Sonora, Mexico. Sampled localities represent the full range of the species and include all formally recognized subspecies (Fig.1). A list of all tissues and their associated data (e.g. museum and/or field number, collection site, and GPS coordinate information) is given in Table S1 (Dryad Digital Respository, doi:10.5061/dryad.77rf2). All necessary permits were obtained for our field efforts in California, Arizona, and Nevada: (1) Arizona permits issued by the Arizona Game and Fish Department (SP613877, SP802036, SP572402, SP755971, SP586491, SP711106), California permits issued by the California Department of Fish and Wildlife (SCP000838, SCP003850, SCP000297, SCP006488, SCP003696, SCP004186), Nevada permit issued by the Nevada Department of Wildlife (S33762), and permits issued by the National Park Service (JOTR 25A0 9–07, JOTR−2005−SCI−0024). All other tissues were obtained from museum and university loans, and the following abbreviations were used for these collections: Arizona State University (ASU); Royal Ontario Museum (ROM); San Diego Natural History Museum (SDSNH); San Diego Natural History Museum Tissue Series (SDField); University of Arizona, (UAZ); and University of Texas, Arlington (UTA). This study did not involve threatened, endangered, or protected species. This study was approved by the Western Ecological Research Center Animal Care and Use Committee in association with the University of California, Davis. Non-destructive tissue sampling techniques (i.e., drawn blood, tail-tips, and salvaged specimens) were used to obtain tissue for DNA extraction. Whole genomic DNA was extracted from tissues using Qiagen DNeasy extraction kits (Qiagen Inc., Valencia, CA, USA).

### mtDNA data collection

We sequenced portions of the mitochondrial 16S rRNA and ND1 genes (total of 1094 bp) for 140 individuals of *C. occipitalis* and three Sonoran shovel-nosed snakes (*C. palarostris*) for use as an outgroup taxon in phylogenetic analyses. We also included the 80 mtDNA sequences generated from our previous study ([37], Genbank numbers: EU280331 – EU280410). The total number of sequences used for mtDNA analyses was 223. Primer sequences and PCR protocols are described in [37]. We purified and directly sequenced PCR products on an ABI 3100 capillary system. We edited and aligned sequences using Sequencher 5.0.

### Microsatellite data collection

Microsatellite library construction was performed in the Sequencing and Genotyping Facility at Cornell University Core Laboratory Center (CLC) using standard development techniques modified from [38]. We used the program msatcommander [39] to scan the FASTA file generated from the 454-automated DNA sequencer (Roche) run for all dimeric, trimeric, tetrameric, and pentameric microsatellites.

We tested 32 loci for variability using six individuals from Arizona and California collection sites. Of the 32 loci, we selected 11 that were variable, amplified consistently, and yielded reliable genotyping scores. We divided these loci into three groups and simultaneously amplified 3–4 loci within each group using a Qiagen multiplex PCR kit in 10 µL reactions containing 5 µL of Qiagen multiplex PCR Master Mix, 1 µL primer mix (containing 2 µM of each primer), 1 µL Q-solution and 2 µL of RNase-free water. Amplified products were run on an ABI 3100 and performed in the CSUPERB Microchemical Core Facility at San Diego State University. We used GENE-MARKER v1.90 (SoftGenetics) to edit the raw allelic data and score allele sizes. We used MICROCHECKER [40] to test for the presence of null alleles and scoring errors. We genotyped 258 individuals and reanalyzed 10% of individuals at all loci.

### Mitochondrial lineage estimates

We used BEAST v1.8.0 [41] to simultaneously estimate phylogenetic relationships and divergence time estimates among mtDNA sequences from a total of 223 individual snakes (nexus file, Dryad Digital Respository, doi:10.5061/dryad.77rf2). We partitioned the data and used the best-fitting models of sequence evolution following Wood *et al*. [37]. Because adequate fossil evidence was lacking for *Chionactis* or closely related sister species, we did not use fossil evidence to calibrate our clock-based analyses. We instead employed fixed substitution rates for the ND1 and 16S rRNA flanking regions that were estimated for other squamates (ND1: 1.39 × 10^−2^ substitutions/site/million years; flanking region: 4.92 × 10^−3^ substitutions/site/million years) [42] to provide lineage age estimates within shovel-nosed snakes. Substitution rates were set in BEAUti v1.8.0 [41] using normal priors, with standard deviations reflecting uncertainty in the estimate. Preliminary analyses were conducted to test the assumption of clock-like evolution and were confirmed using the Bayes Factor test [43] and implemented in Tracer 1.5 [44]. We conducted four independent runs in BEAST using a strict clock model and a coalescent constant size tree prior. We obtained posterior distributions of model parameters and genealogies by sampling from the Markov chain Monte Carlo (MCMC) posterior distribution using four chains every 1000th generation for a total of 20 million generations (first 40% of samples were discarded as burnin). We assessed convergence by visually inspecting the cumulative posterior probabilities of split frequencies using the program awty [45]. We used the maximum clade credibility tree and posterior probabilities (*Pp* ≥ 0.95) to infer mtDNA relationships and evaluate support for phylogenetic lineages within *C. occipitalis*.

### Microsatellite diversity and cluster estimates

Genetic diversity was assessed at each microsatellite locus and genetic cluster by estimating the observed heterozygosity (*H*_O_), expected heterozygosity (*H*_E_), number of alleles (*N*_A_), and allelic richness (*A*_R_). These genetic indices were calculated using GENALEX v6.41 [46]. We tested for linkage disequilibrium between all pairs of loci using Fisher's exact tests implemented in GENEPOP on the web [47], [48], and the level of statistical significance (α  =  0.05) was adjusted using the Bonferroni correction [49].

We used two Bayesian genotypic clustering analyses to infer discrete genetic clusters within the dataset at two different spatial scales (microsatellite data file, Dryad Digital Respository, doi:10.5061/dryad.77rf2). First, we conducted a range-wide analysis of population structure using structure version 2.3.2 [50], a nonspatial algorithm that is routinely used for identifying the number of genetic clusters in microsatellite datasets. Second, a fine-scale analysis was conducted on collection sites sampled across Arizona to detect spatial patterns where *C. o. annulata* and *C. o. klauberi* supposedly intergrade. For this analysis, we used the clustering algorithm implemented in geneland version 3.3 [51] because it can take spatial information into account to produce inferences of genetic structure and boundary locations based on the geographic distribution of individuals. Both programs group individuals into the most likely number of clusters (*K*) that maximizes the within-cluster Hardy-Weinberg and linkage equilibria.

For the structure analyses, the number of clusters (*K*) that putatively best explains the dataset is inferred from the posterior probability distribution of the data given the range of *K* specified by the user, P(*X*|*K*). We ran the admixture model with an uncorrelated frequency model. We inferred the number of clusters (*K*) by comparing the results from the mean lnP(D|*K*) score against the K_max_ (i.e. where the lnP(D|*K*) curve plateaus) and the Δ *K* criterion [52]. We also hierarchically tested for substructuring within clusters by performing subsequent structure analyses within each inferred cluster [53]. For all structure analyses, we estimated the probability of one through 10 clusters (*K*) using 1,000,000 iterations of the MCMC algorithm following a burn-in of 500,000 iterations, with each run replicated ten times to check for the consistency across the Markov chains.

For geneland analyses, all parameters (including the number of clusters, *K*) are co-estimated simultaneously by the MCMC algorithm. Once completed, the most probable number of clusters and their geographic boundaries are inferred from the highest average posterior probability density (PPD) of genetic clusters. We followed the recommendations given by Guillot *et al*. [51] that data analyses begin with the uncorrelated frequency model. Although the correlated model can be more powerful at detecting subtle differentiation of genetic structure, it also can be more prone to depart from model assumptions (e.g., presence of isolation-by-distance) that are present in our dataset. We performed five independent runs of *K*  =  1–5 with 10,000,000 MCMC iterations and a thinning of 250. Coordinates from each individual were used to run the spatial model and the potential error for spatial coordinates was set at 3 m. Post-processing of the MCMC outputs with the highest PPD followed a burn-in of 10,000 in order to obtain posterior probabilities of cluster membership for each individual.

We used *F*_ST_ estimates and isolation-by-distance (IBD) analyses as secondary methods to summarize microsatellite variation and to identify significantly differentiated genetic clusters inferred from the assignment tests. Pairwise genetic distances among clusters were based on allele frequency differences using *F*_ST_ following [54] and were implemented in GENALEX v6.41. To test for genetic isolation by distance, we compared pairwise matrices of geographic and genetic distance using Mantel tests for matrix correlation [55], with significance assessed by 5,000 randomizations of the genetic distance matrix. We calculated the individual-based genetic distance *â* developed by Rousset [56] between all pairs of individuals within each of the three clusters using the program GENEPOP on the web. In each analysis, we examined untransformed and log transformed axes, and report the combination that exhibited the highest correlation coefficient. IBD was examined for all sites and across three clusters identified with geneland (see results) that span the geographic range of *C. o. annulata* and *C. o. klauberi*. Because clustering approaches can sometimes mistake IBD for hierarchical population structure [57], we also performed a series of partial Mantel tests to assess whether the clusters identified by geneland could be explained by IBD. We accomplished this by testing the association between genetic distances and cluster membership with geographic distances treated as a covariate. All IBD analyses were performed in IBDWS 3.21 [58].

### Microsatellite genetic isolation estimates

We used the population migration rate (2*N_e_m*), which is the effective rate at which genes enter a population, as a criterion for defining the relative strength of genetic isolation between the range-wide clusters identified using structure [15]. We used the isolation-with-migration model implemented in the program IMa2 [59], [60] to estimate effective population migration rates. We specified the topology of the population tree that is required for IMa2 analysis based on the mtDNA tree [Mojave, (Colorado, Sonoran)]. We used all 11 loci to estimate demographic parameters and applied the stepwise mutation model and an inheritance scalar of 1.0 to each locus. We assumed a generation time of 3 years for *C. occipitalis* to obtain a measure of migration on a scale of generations, which was based on demographic observations of a similar fossorial species, *Chilomeniscus straminus* (P. C. Rosen, personal communication). We used the state of the Markov chain from a long run using final priors to seed three separate runs (10,000 genealogies saved from each), after a series of initial runs to determine the most appropriate search parameters that maximized mixing.

For each analysis, we used the following settings for the prior distributions on population parameters: 160 chains with a geometric heating scheme (g1  =  0.99 and g2  =  0.5), maximum scalars for theta (*q_0_*  =  40; *q_1_*  =  40; *q_2_*  =  40; *q_3–4_*  =  160), maximum migration prior value (*m*  =  3), and maximum time of population splitting (*t*  =  15). A total of 100,000 steps were retained with a step length of 20 and the first 150,000 steps discarded as burn-in. We ordered the relative strength of the 2*N_e_m* estimates following recommendations of [15] and [61]: strong isolation when 2*N_e_m* ≤ 1, moderate isolation when 1 < 2*N_e_m* ≤ 5 and weak when 5 < 2*N_e_m* ≤ 25.

## Results

### MtDNA lineages

**Figure 2:**
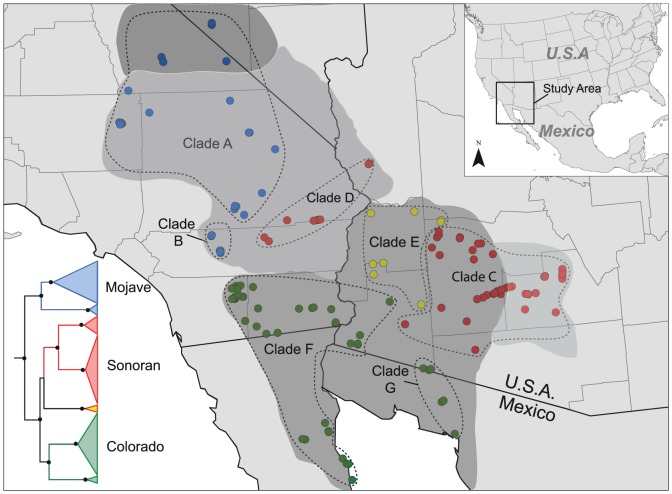
Phylogeny for the western shovel-nosed snake (*Chionactis occipitalis*) based on partitioned Bayesian analysis of mitochondrial DNA sequence data (16S rRNA and ND1 genes) and the geographic distribution of the major lineages (Mojave lineage in blue, Sonoran lineage in red, and Colorado lineage in green). The geographic distribution of clades within each lineage are outlined with a dashed line, clade E (yellow clade and dots) was routinely placed as sister to the Sonoran lineage, but posterior probabilities supporting this relationship were weak (*Pp* < 0.50). Black circles at nodes represent Bayesian posterior probabilities of ≥ 0.95. See Figures S1-3 for more detail within each mtDNA lineage.

The mtDNA Bayesian analyses yielded a well-resolved phylogeny. Most notably, *C. occipitalis* was composed of three geographically distinct mtDNA lineages that were similar to Wood *et al.* [37], although the increased geographic sampling in this study provided added resolution of clade boundaries and increased posterior probability support for a third lineage (Fig. 2; Fig. S1-3). The northernmost lineage, hereafter called the ‘Mojave lineage’, was composed of two well supported clades. Individuals belonging to clade A occupied the range of *C. o. occipitalis* and *C. o. talpina* throughout the Mojave Desert, with the exception of samples taken from eastern Mojave Desert in California and Arizona (see below). Individuals of clade B occurred farther south in the range of *C. o. occipitalis* and were restricted to the Coachella Valley in California. A second lineage, hereafter called the ‘Sonoran lineage,’ was composed of two geographically disjunct clades (clades C and D). Individuals of clade C were primarily located in the Sonoran Desert of central Arizona encompassing the range of *C. o. klauberi* and the northeastern range of *C. o. annulata*. Clade D encompassed the range of *C. o. occipitalis* within the southeastern portion of the Mojave Desert in California and Arizona. A third clade (clade E) was routinely placed as sister to the Sonoran lineage, but posterior probabilities supporting this relationship were weak (*Pp* < 0.50). This clade was located in the Sonoran Desert of western Arizona, partially encompassed the ranges of *C. o. annulata* and *annulata-klauberi* ‘intergrades’, and was geographically nested in between clade C and D. Finally, the southernmost lineage, hereafter called the ‘Colorado lineage,’ occupied the southwestern portion of the range of *C. o. annulata* and was distributed in the lower Colorado River subdivision of the Sonoran Desert in California and Baja California (clade F), and southwestern Arizona and Sonora, Mexico (clade G).

Our Bayesian clock estimates of divergence indicate that diversification of the major lineages within *C. occipitalis* occurred in the Pliocene to early Pleistocene (Table 1), which are consistent with molecular clock estimates reported by Wood *et al*. [62]. The Mojave lineage diverged from the Colorado and Sonoran lineages in the Pliocene (3.3 Ma, 95% HPD 3–4 Ma), followed by further divergence of Colorado and Sonoran lineages in the late Pliocene and early Pleistocene (3.0 and 1.8 Ma respectively).

**Table 1:** Divergence time estimates from BEAST analyses.

MRCA	Coalescent age estimate
Mojave lineage	3.3 (2.6–4.2)
Colorado lineage	3.0 (2.4–3.6)
Sonora lineage	1.8 (1.3–2.4)
*Chionactis*	7.3 (5.6–9.3)

Reported values are the time to most recent common ancestry (MRCA; means reported in millions of years before present and followed by 95% HPDs).

### Genetic structure inferred from microsatellites

Allelic richness for each microsatellite locus genotyped across the 258 individuals ranged between 6.1 and 15.1 (9.2 on average), and observed heterozygosity for each locus ranged from 0.419 and 0.812, with an average value of 0.665 for all loci (Table 2). There were no pairs of loci with significant linkage disequilibrium after Bonferroni correction (P  =  0.0009).

**Table 2:** Summary of repeat motif, observed heterozygosity (*H*_O_), and expected heterozygosity (*H*_E_), total number of alleles (*N*_A_), and allelic richness (*A*_R_) for each microsatellite locus.

Locus	Motif	*H*_O_	*H*_E_	*N*_A_	*A*_R_
PEN160	(AATGG)^6^	0.689	0.772	15	8.40
TRI176	(AAC)^14^	0.606	0.722	13	7.78
TET1713	(ATCT)^13^	0.756	0.849	19	9.12
TRI1164	(AAC)^19^	0.812	0.856	16	10.72
PEN5400	(AATAG)^9^	0.744	0.892	17	10.76
TRI222	(ACT)^14^	0.525	0.814	11	8.42
TRI1925	(AGG)^9^	0.419	0.522	12	6.14
TRI199	(AAC)^11^	0.61	0.701	9	6.86
TET193	(CTTT)^13^	0.705	0.874	19	10.16
TRI2219	(ACT)^10^	0.75	0.745	10	7.33
TET1847	(AGAT)^11^	0.703	0.903	31	15.08

**Figure 3:**
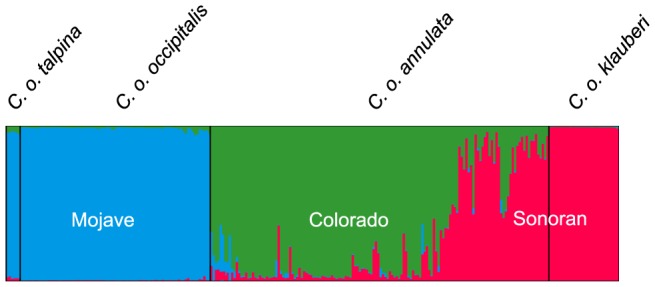
Assignment probabilities based on the structure analysis (*K*  =  3). **a) Each individual is represented by a single column with membership assignment probabilities for each of the three clusters (*K*) noted as the relative proportion of each color (blue, green, and red represent the Mojave, Colorado, and Sonoran clusters, respectively).** The vertical black bars represent the *a priori* subspecies assignment for each individual that was used in the analysis.

**Figure 4:**
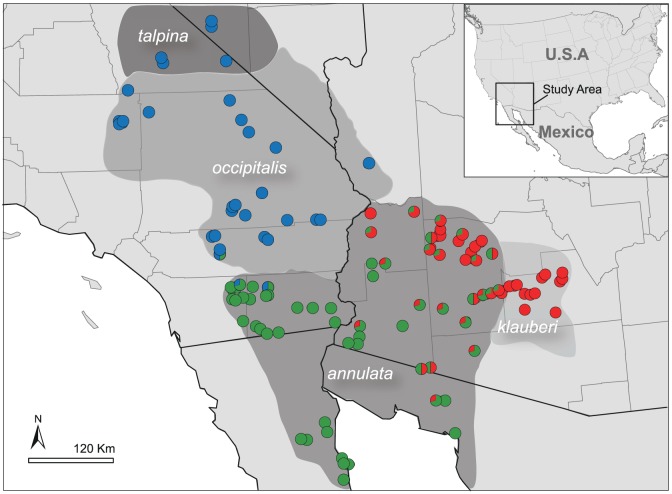
Range-wide geographic distribution of the three clusters inferred by structure (*K*  =  3) overlaid on the subspecies distributions. Circles on the map are colored proportional to their posterior probability assignment to each of the three clusters.

At the range-wide spatial scale, structure analyses strongly indicated a sharp plateau at *K*_max_ =  3 as an approximate estimate for *K* under the admixture model. This result was corroborated by the Δ *K* criterion (Fig. S4). On the basis of *K*_max_ =  3, cluster assignments for each sample and the geographic distribution of each cluster, hereafter referred to as the Mojave, Colorado, and Sonoran clusters, are depicted in Figures 3 and 4.

The Mojave cluster encompassed the range of *C. o. occipitalis* in California and Arizona as well as the range of *C. o. talpina*, and the proportion of each individual's genome assigned to this cluster was fairly exclusive (i.e., ≥ 0.95). Unlike the mtDNA patterns, *C. o. occipitalis* from the eastern Mojave Desert in California and Arizona were grouped with other geographically proximate individuals of *C. o. occipitalis* rather than clustering with individuals of *C. o. annulata* in the Sonoran Desert as inferred using the mtDNA data (see mtDNA clade D). The Colorado cluster encompassed the range of *C. o. annulata* in California, Arizona, and Mexico and portions of the previously reported intergrade zone. Finally, the Sonoran cluster occupied the presumed range of *C. o. klauberi*, portions of the previously reported intergrade zone, and a small portion of the range of *C. o. annulata* in west-central Arizona. Admixture among the Colorado and Sonoran clusters was apparent in the vicinity of the reported intergrade zone. Individual snakes sampled throughout this region tended to exhibit lower probability assignments (0.5 ≤ P ≤ 0.7; see Fig 4), a pattern consistent with contemporary gene flow between *C. o. annulata* and *C. o. klauberi*. Within each of the three clusters, we conducted additional structure runs to look for evidence of further genetic structure, but additional clusters were not supported.

**Figure 5:**
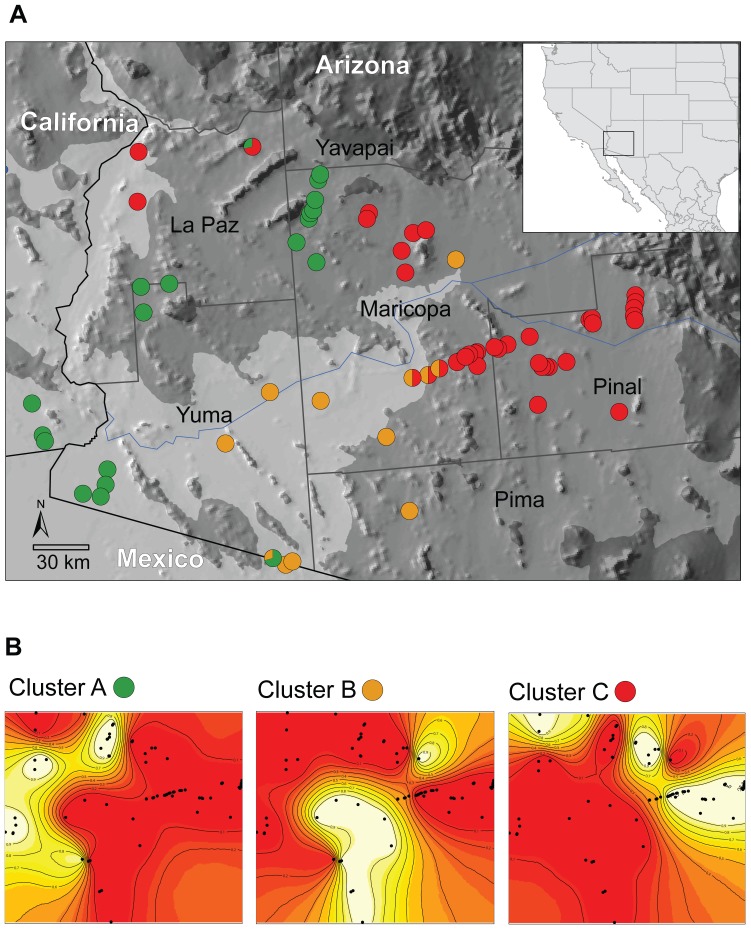
Spatially-explicit estimate of population structure across Arizona based on the geneland analysis. **a**) Map of Arizona and the geographic distribution of sampled individuals colored according to the probability of assignment within each cluster identified using geneland (green, orange, and red circles represent individuals assigned to Clusters A, B, and C, respectively). The shading indicates elevations below 300m (light grey), between 300 and 1000m (grey) and above 1000m (dark grey). **b**) Geneland probability contour maps for the three clusters across Arizona. The highest posterior probabilities are in white (p ≥ 0.9) and the lowest are in red (p ≤ 0.1) – contour lines within each map depict the spatial change in population assignment probability.

We restricted our geneland analyses to collection sites sampled across Arizona to detect fine-scale spatial patterns and boundary locations where *C. o. annulata* and *C. o. klauberi* intergrade. Taking into account the spatial location of genotyped samples across Arizona, the MCMC analysis of genetic structure using geneland placed greater than 99% of the posterior density on *K* =  3 (Fig. S5). The spatial depictions of cluster membership and posterior probability contours are presented in Figure 5. The assignments across Arizona were geographically similar with the results obtained using structure. However, an additional cluster was identified and boundary locations for each were marked by sharp posterior probability contours. The most notable feature of the geneland analysis was the spatial distribution of cluster B. This cluster was primarily composed of individuals that were of admixed assignment according to the structure analysis and encompassed the reported intergrade zone between *C. o. annulata* and *C. o. klauberi*. The spatial distribution of cluster C included the same localities as the Sonoran cluster identified by the structure analysis (Pinal, Maricopa, and northern La Paz Counties; Fig. 5). However, in northwestern Maricopa County, several individuals with admixed structure assignments were assigned to cluster A and one to B, all with high posterior probabilities. Other similarities between the cluster analyses included snakes sampled along the west-to-east transect in Maricopa and Pinal Counties. In both analyses these samples exhibited increasing posterior probability assignments from one cluster to another that coincides with the presumed transition from *C. o. annulata* to *C. o. klauberi*.

### Genetic diversity and differentiation using microsatellites

Measures of genetic diversity did not vary considerably across the three structure clusters. We provide general summary statistics, including number of samples, average number of alleles, heterozygosity, and fixation index in Table 3.

**Table 3:** Genetic variability of microsatellite loci for each cluster identified using structure.

Cluster	*N*	*A*_RCor_	*H*_O_	*H*_E_	*F*
Mojave cluster	88	10.2	0.64	0.75	0.15
Colorado cluster	105	11.9	0.68	0.83	0.17
Sonoran cluster	71	11.2	0.65	0.75	0.12
Total	264	12.6	0.66	0.78	0.15

Notations are as follows: number of samples (*N*), average number of alleles (*A*_RCor_), observed heterozygosity (*H*_O_), expected heterozygosity (*H*_E_), and fixation index (*F*) for all microsatellite loci.

We used pairwise *F*_ST_ estimates as an additional method to test the significance of genetic differences between the identified clusters. Allele frequencies were significantly different between the clusters identified by structure (Table 4). The highest among cluster pairwise *F*_ST_ estimates were found between the Mojave and Sonoran clusters (*F*_ST_  =  0.141) and lowest pairwise comparison was between the Mojave and Colorado clusters (*F*_ST_  =  0.054). We detected a significant isolation-by-distance pattern for all collection sites combined (r  =  0.346, P ≤ 0.001) and for the three geneland clusters across Arizona (r  =  0.385, P ≤ 0.001). However, partial Mantel tests revealed a significant association between clusters and genetic distance (r  =  0.116, P ≤ 0.001) after correcting for the geographical distances. This result suggests allele frequency differences between clusters are not due to geographic distance alone.

**Table 4:** Pairwise *F_st_* among clusters of *Chionactis occipitalis* identified by structure, all estimates were significant after Bonferroni correction.

	Cluster 1	Cluster 2	Cluster 3
Mojave cluster	–		
Colorado cluster	0.054	–	
Sonoran cluster	0.141	0.078	–

The IMa2 genetic isolation estimates detected asymmetric rates of gene migration between clusters obtained from structure analyses (Table 5). Moderate values of genetic isolation were detected between Mojave and Sonoran clusters, with greater gene migration (2*N_e_m*) occurring from the Sonoran cluster to the Mojave cluster compared to the opposite direction. Strong to moderate genetic isolation was detected between the Colorado and Sonoran clusters, and higher gene migration was inferred from the Sonoran cluster into the Colorado cluster. We detected negligible variation among 2*N_e_m* values estimated between the Mojave and Colorado clusters, where gene migration was strongly restricted in both directions.

**Table 5:** Population migration rates (2*Ne*M) estimated from IMa2.

	M into S	S into M	M into C	C into M	S into C	C into S
Point estimate	**1.91**	**4.43**	**0.78**	**0.57**	**4.08**	**0.00**
95%Lo	0.77	2.75	0.20	0.28	3.24	0.00
95%Hi	2.88	5.84	1.23	1.11	5.29	0.53
Relative isolation	*moderate*	*moderate*	*strong*	*strong*	*moderate*	*strong*

Point estimates of migration (in demographic units) are reported as the rate at which genes from one cluster enter another cluster over time. M, C, and S refer to the three clusters (Mojave, Colorado, and Sonoran, respectively) recovered from the structure analysis. Relative isolation was ranked based on recommendations from [10] and [56].

## Discussion

### Phenotypic subspecies correspondence with genetic structure

In this study, we used both mitochondrial DNA and 11 microsatellite loci to further assess whether patterns of population genetic structure are concordant with the spatial structuring of phenotypic variation that originally led to the subspecies descriptions within *Chionactis occipitalis*. This significantly expanded geographic sampling and the addition of nuclear loci, allowed us to better characterize the genetic structure within *C. occipitalis*. Our study did not find support for *C. occipitalis* subspecies as exclusive genetic groups. Patterns of discordance between the phenotypic and genotypic boundaries were detected in areas where *C. o. occipitalis*, *C. o. annulata*, and *C. o. klauberi* are presumed to be in contact. The only exception to this pattern was *C. o. talpina*, which was entirely nested within *C. o. occipitalis* on the basis of both mtDNA and microsatellite datasets.

Differences in the spatial distribution of genetic and phenotypic variation are not unexpected when the boundaries of divergent lineages are parapatric. For instance, even though lineages may have diverged to the point of having some diagnosable differences (both phenotypic and genetic), these lineages may not be sufficiently reproductively isolated from each other to prevent gene flow in secondary contact. Although gene flow is often a homogenizing force [63], [64], instances of population (or species) divergence despite gene flow exist in snakes and other taxa [65]–[69]. Such admixture can create fuzzy boundaries along these zones of contact. Therefore, criteria that demand perfect overlap between phenotypic and genetic divergence patterns may not be realistic for parapatric entities, whether subspecies or species.

These results highlight the difficultly in delimiting taxonomic boundaries within lineages that are evolving under a time-dependent, continuous process (e.g., species – subspecies boundary) [70]. Although we showed a lack of genetic exclusivity among the subspecies of *C. occipitalis*, there were a large proportion of individuals within *C. o. occipitalis, C. o. annulata*, and *C. o. klauberi* that were assigned to the Mojave, Colorado, and Sonoran clusters (Fig. 4), respectively. Contact between *C. o. occipitalis* and *C. o. annulata* is presumed to occur along the Mojave and Sonoran Desert ecotone, with putative intergrades following the crests of the Little San Bernardino and Chocolate Mountains in eastern Riverside and Imperial Counties in California [29]. These boundaries are largely consistent with our multi-locus genetic data (Fig. 4). Klauber [29] noted the only exception to this division lies within the Coachella Valley in the northern Colorado Desert of California, the southernmost region occupied by *C. o. occipitalis*. He suggested that *C. o. occipitalis* may have accessed the Coachella Valley through the low valley passes within the Little San Bernardino Mountains where it eventually meets (according to phenotypic patterns) with *C. o. annulata* along the northwestern region of the Salton Sea at the Imperial-Riverside county line. Assignment probabilities of *C. o. annulata* individuals within the northern Colorado Desert are consistent with some genetic admixture in this region. Although our gene migration estimates indicate strong isolation between Mojave (*occipitalis*) and Colorado (*annulata*) clusters, these estimates are greater than zero suggesting that complete isolation between them has not been achieved.

We also found that *C. o. klauberi* and *C. o. annulata* were not genetically exclusive. The cluster containing all samples of *C. o. klauberi* (Sonoran cluster & cluster C according to structure & geneland, respectively) also included samples within the putative intergrade zone and a few *C. o. annulata* from northern La Paz County in Arizona. The key morphological characteristic used to diagnose *C. o. klauberi*, dark maculations infused within the red secondary bands that contact dorsally [29], are not present in all individuals forming this cluster, but this character likely increases in frequency from west to east throughout this cluster. Although phenotypic variation was not examined rigorously here, the presence of dark maculations were observed in most individuals assigned to the Sonoran cluster, but were primarily present laterally rather than connecting along the dorsum. However, Klauber [29] observed that the dark maculations only contact dorsally in about 50% of *C. o. klauberi*. He further noted several localities with “*klauberi* influences”, in particular “the Wickenburg area” and “east of Aguila” in northwestern Maricopa County, that corroborate well with the inferred cluster boundaries (Fig. 4 & Fig. 5a), which suggests that the western boundary of *C. o. klauberi* may be more extensive than previously assumed.

Although previous interpretations of phenotypic variation had presumed a broad intergrade zone between *C. o. annulata* and *C. o. klauberi* [36], the genetic data identified specific locations of genetic admixture that are primarily restricted within central Arizona (Fig 5, cluster B). From our sampling scheme, two regions of gene migration are apparent and merit further investigation: (i) within northwestern Maricopa County along the Gila River valley through Buckeye valley to the Hassayampa River valley, and (ii) within southwestern Maricopa County along the Gila River valley west of the Maricopa Mountains (Fig 5). Additional research quantifying both phenotypic and genetic variation should help to better understand whether the zone of contact results in uniformly admixed populations or whether divergence with gene flow is maintained between two distinctive phenotypes.

### Taxonomic status

Previous authors [36] speculated whether the subspecies within *C. occipitalis* provided any meaningful understanding of the evolutionary history of this species (i.e., whether they serve as useful taxonomic surrogates for evolutionary lineages). The multilocus perspective presented here (mtDNA & microsatellites), provided evidence for at least three divergent groups that have broadly concordant boundaries with three of the phenotypically recognized subspecies (*occipitalis*, *annulata*, and *klauberi*). Because taxonomy should be consistent with the evolutionary history of a group, it is possible that these genetic groups represent independent evolutionary lineages and therefore constitute distinct species (following criteria outlined in the evolutionary and general lineages species concepts) [1], [70], [71]. Among these three, *klauberi* exhibited the most discordance between the spatial distribution of genetic and phenotypic patterns, but this discordance may simply reflect the recency of its divergence (compared to divergence estimates between *occipitalis* and *annulata*), asymmetric gene flow along the zone of contact, and ambiguous delineation of the western phenotypic boundary. Therefore, some discordance within this geographic region would be expected. Nonetheless, we feel that the current phenotypic and genetic data available for *klauberi* at this time allows neither definitive acceptance nor rejection of species status for the lineage. Therefore, we propose the most advantageous and conservative strategy is to recognize two species in the group that conform to the divergent and phenotypically distinct Mojave and Colorado-Sonoran clusters: the Mojave shovel-nosed snake, *Chionactis occipitalis* (type locality, San Bernardino County, California) [72] and the Colorado Desert shovel-nosed snake, *C. annulata* (type locality, Imperial County, California) [73], [74]. Previously reported color pattern variation along with the genetic variation can be used to diagnose these two species hypotheses (Table 6). In general, *C. occipitalis* possess more brown primary crossbands and no secondary red crossbands, while *C. annulata* have fewer black primary crossbands and possess red-to-orange secondary crossbands [29]. We also recommend retaining *annulata* and *klauberi* as subspecific designations within *C. annulata* in keeping with the genetic structure that was recovered across Arizona, while placing *talpina* in synonomy with *C. occipitalis*. In this framework, maintenance of the subspecific nomenclature within *C. annulata* provides putative species hypotheses that can be examined in more detail, and provides important taxonomic handles for conservation policy and wildlife management agencies.

**Table 6:** Summary of each diagnostic characteristic for the *Chionactis* species complex based on the multi-locus dataset and Klauber [29]: values (n) below each species/subspecies name refer to the number of snakes for which both genetic & color pattern characters were collected, percentages represent the proportion of snakes that exhibited each phenotypic character.

	C. palarostris (n = 3)	C. occipitalis (n = 35)	C. annulata (n = 61)	C. a. annulata (n = 28)	C. a. klauberi (n = 33)
ND1 % sequence divergence from *C. palarostris*	−	*7.5*	*7.4*	−	−
MtDNA lineage	−	Mojave + clade D	Colorado + Sonoran (excluding clade D)	−	−
Nuclear microsatellite cluster	−	Mojave	Colorado + Sonoran	Colorado	Sonoran
Primary crossband color	Black	Brown	Black	−	−
Red secondary crossband	Present (100%)	Absent (91%)	Present (96%)	−	−
Number of primary crossbands*	usually < 23 (100%)	usually ≥ 45 (82%)	usually < 44 (85%)	−	−
Head crescent engages posterior edge of frontal scale	−	usually ≥ 25% (80%)	usually just tip or none (70%)	−	−
Maculations	−	−	−	usually absent (72%)	usually present (97%)
Proportion of maculations that occur in center of scales	−	−	−	38%	88%

*taken as a combination of the number of dorsal primary crossbands on the body (not including the tail bands) plus the number of unmarked ventral band positions, Klauber [29].

### mtDNA vs. microsatellite variation

Because the genetic structure results differed slightly between marker types (mtDNA vs microsatellites), it is important to note where they differ and what these data reveal about subdivision within this species complex. Phylogenetic analysis of the mtDNA sequencing data revealed strong support for regionally distinctive lineages that sorted by desert basins (i.e. Mojave, Colorado, and Sonoran Deserts), with some overlap of lineages extending across desert boundaries. Likewise, our range-wide assignment tests based on microsatellite data confirmed the presence of at least three geographically cohesive clusters. The spatial discordance among gene tree lineage and cluster boundaries was narrow and occurred along two zones of contact. First, *C. occipitalis* mtDNA haplotypes from the eastern Mojave Desert (mtDNA clade D) were nested within the mtDNA Sonoran lineage, but the microsatellite data assigned these same samples with high probability to all other *C. occipitalis* samples within the Mojave Desert. In the same way, haplotypes of *C. a. annulata* from west-central Arizona (clade E) formed a well-supported mtDNA clade, but could not be grouped with confidence to either the Colorado or Sonoran lineages. However, nuclear genotypes of these same samples were assigned to the genetic cluster containing the majority of all other *C. a. annulata* samples. Thus, it appears that phylogenetic relationships based on mtDNA for clades D and E are misleading and suffer from introgression at lineage boundaries.

Wood *et al.* [37] noted the discordance between mtDNA and phenotypic patterns in these two regions, and suggested incomplete lineage sorting or contemporary gene flow as potential causes for the lack of agreement. Based on the results of this study, the likely cause of discordance is recent gene flow. Our mitochondrial divergence date estimates suggest that the recovered genetic groups may be quite old, having diverged approximately 1.8–3.3 Ma. Considering the age of diversification and geographic cohesiveness of the mitochondrial lineages, past mitochondrial gene flow, if it occurred at all, did not spread far beyond the contact zone. Second, results from our IMa2 analysis confirmed contemporary gene flow between the three clusters (where higher migration rates were observed from the Sonoran cluster into both Mojave and Colorado clusters). Because the sequencing analyses were conducted on a single mitochondrial locus, whereas the microsatellite analyses incorporated data from 11 different loci, our mtDNA patterns are likely more susceptible to locus-specific stochastic effects of gene flow at clade boundaries than the microsatellite data.

### Phylogeography

The southwestern deserts of North America are marked by phylogeographical breaks that result in distinctive genetic groups within species occupying this region. By comparing the phylogeographical signature of co-distributed taxa, Wood *et al.* [62] were able to identify broad spatial divisions that were associated with geographical barriers such as the Colorado River and the Mojave and Sonoran Desert ecotone. Division of lineages was associated with diversification events within the late Pliocene or older, but some discontinuities within species also corresponded to more recent events within the Pleistocene. With regard to our study, the recovered lineages have apparently existed in the southwestern deserts for a long period of time (≥ 3Ma; Table 2), only the Sonoran lineage had estimates that encompassed the Pleistocene. Such deep genetic structuring may indicate lineage diversification due to long standing barriers to gene flow that have since relaxed, allowing for possible secondary contact. Alternatively, the observed genetic structuring in this group may also indicate regional diversification driven by an adaptive response to physical and biotic factors that differ across the range. The boundary between *C. occipitalis* and *C. annulata* is defined by an abrupt transition in elevation, temperature, total rainfall, vegetation and terrain features known as the Mojave and Sonoran ecotone [75], [76]. Such environmental gradients, if strong enough, may restrict gene flow [65], [77], [78]. Several other aspects of our analyses are consistent with this hypothesis: (i) genetic differentiation among clusters remained significant, even after accounting for geographic distance among sampling locations, suggesting that some other mechanism besides geographic distance is driving genetic divergence, (ii) previous analyses found that the strongest predictors of genetic divergence among mitochondrial lineages were elevation, temperature and desert basin assignment [37], and (iii) we found asymmetric rates of gene flow between some clusters. While the spatial genetic patterns detailed in this study remain consistent with historical biogeographic reconstructions of the southwestern deserts (reviewed in [62]), the factors mentioned above lead us to believe there may also be more contemporary ecological and landscape barriers at play, equally sufficient to maintain genetic differentiation of the recovered lineages that may be locally adapted to specific desert basin conditions.

## Conclusions

We have used data from a combination of mtDNA sequences and 11 microsatellite loci to study the genetic structure and phylogeography of Western shovel-nosed snakes. We evaluated whether phenotypic subspecies definitions were supported by genetic data, and did not find exact spatial congruence with the original subspecies hypotheses. Despite these results, both mitochondrial and nuclear data sets corroborated similar divergence patterns for at least three groups that are in differential contact along common distributional boundaries with low to moderate levels of gene exchange. Based on the available evidence, we suggested that species-level diversity is underestimated in this group. We proposed that at least two species be recognized, *Chionactis occipitalis* and *C. annulata*. For now, we recommend retention of the names *annulata* and *klauberi* as subspecific designations within *C. annulata* that conform to the patterns of genetic structure (i.e., Colorado and Sonoran clusters, respectively), while placing *C. o. talpina* in synomony with *C. occipitalis*. Whether additional species should be recognized within *C. annulata* will depend on future characterization of morphological and genetic variation within the zone of contact.

Because species require genetic diversity to respond to changing environmental conditions, conservation strategies aimed at protecting regional genetic groups can maximize the species' evolutionary potential and resilience [79], [80]. Given the broad (but not exclusive) geographic cohesion found between the phenotypic and genetic variation in *C. a. annulata* and *C. a. klauberi*, continued recognition of each may assist in preserving the potential for future evolutionary change within this species. Further, these results may be particularly valuable for regional conservation planning in south-central Arizona. In addition to recovering unique genetic variation primarily within the range of *C. a. klauberi*, we were also able to better quantify the geographic extent of genetic intergradation with *C. a. annulata*. Instead of existing as a gradual cline over a broad area, the zone of intergradation was narrower and existed as steep shifts in allele frequencies. Gene flow between divergent lineages can create novel variation that can facilitate adaptive evolution [81]–[84]. Thus, protection of such diversity may best be achieved by maintaining viable populations within these heterogeneous areas – both ‘pure’ and the hybrid populations [14], [10], [85].

## Supporting Information

Figure S1 **Mojave lineage pruned from the full mtDNA phylogeny, shown in the upper left.** Numbers at nodes represent posterior probability support values for individual clades.
(PDF)

Figure S2 **Sonoran lineage pruned from the full mtDNA phylogeny, shown in the upper left.** Numbers at nodes represent posterior probability support values for individual clades.
(PDF)

Figure S3 **Colorado lineage pruned from the full mtDNA phylogeny, shown in the upper left.** Numbers at nodes represent posterior probability support values for individual clades.
(PDF)

Figure S4 **Results from the mean lnP(D|** ***K*** **) score against the K_max_ and the Δ** ***K*** **criterion of Evanno et al. [72]** **.**
(PDF)

Figure S5 **Posterior density of the number of clusters (** ***K*** **) from the MCMC analysis of genetic structure across Arizona using geneland.**
(PDF)

Table S1 **Tissue samples of** ***Chionactis occipitalis*** **used in this study along with locality information, Dryad Digital Repository, doi:10.5061/dryad.77rf2.**
(XLSX)
