# Supplementary figures and images for: Fuzzy Boundaries: Color and Gene Flow Patterns among Parapatric Lineages of the Western Shovel-Nosed Snake and Taxonomic Implication
Source: PLoS One. 2014 May 21;9(5):e97494. doi: 10.1371/journal.pone.0097494 (PMC4029750; doi:10.1371/journal.pone.0097494)

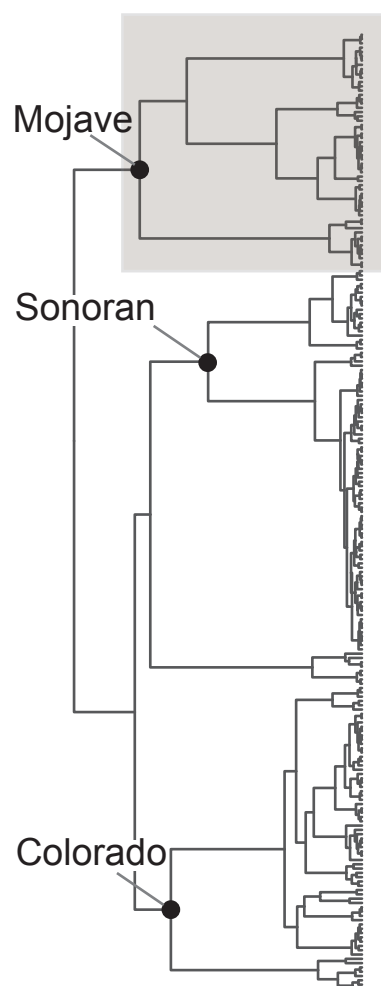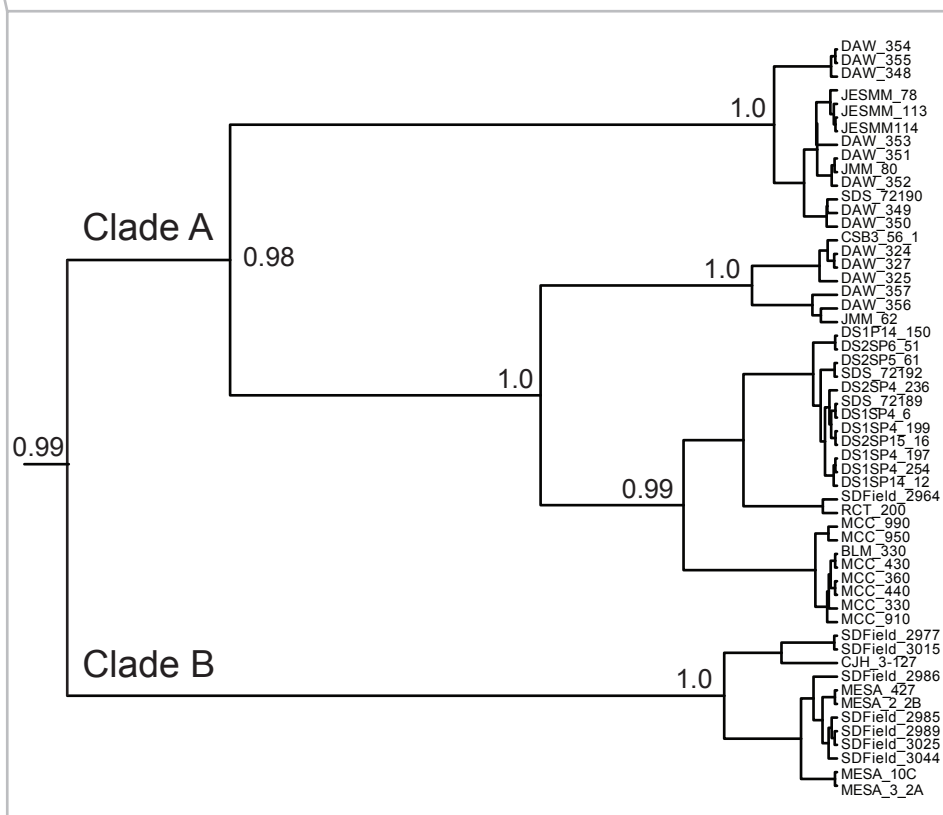

Supplement: Figure S1 — Mojave lineage pruned from the full mtDNA phylogeny, shown in the upper left. Numbers at nodes represent posterior probability support values for individual clades. (PDF) [file pone.0097494.s001.pdf]

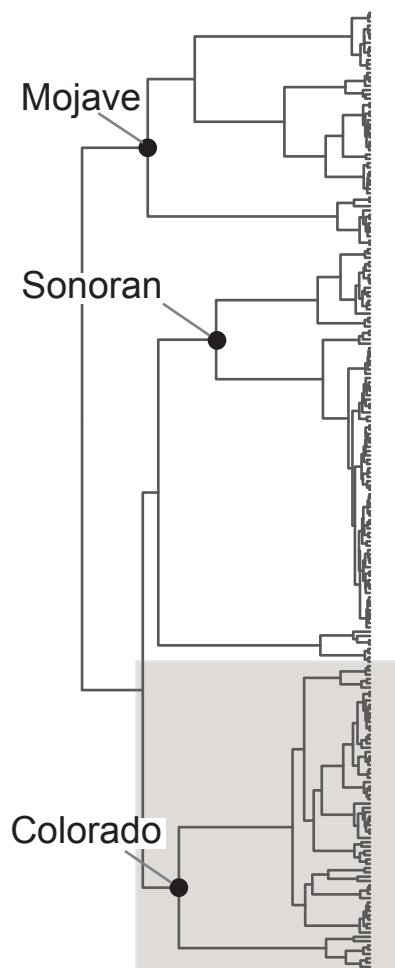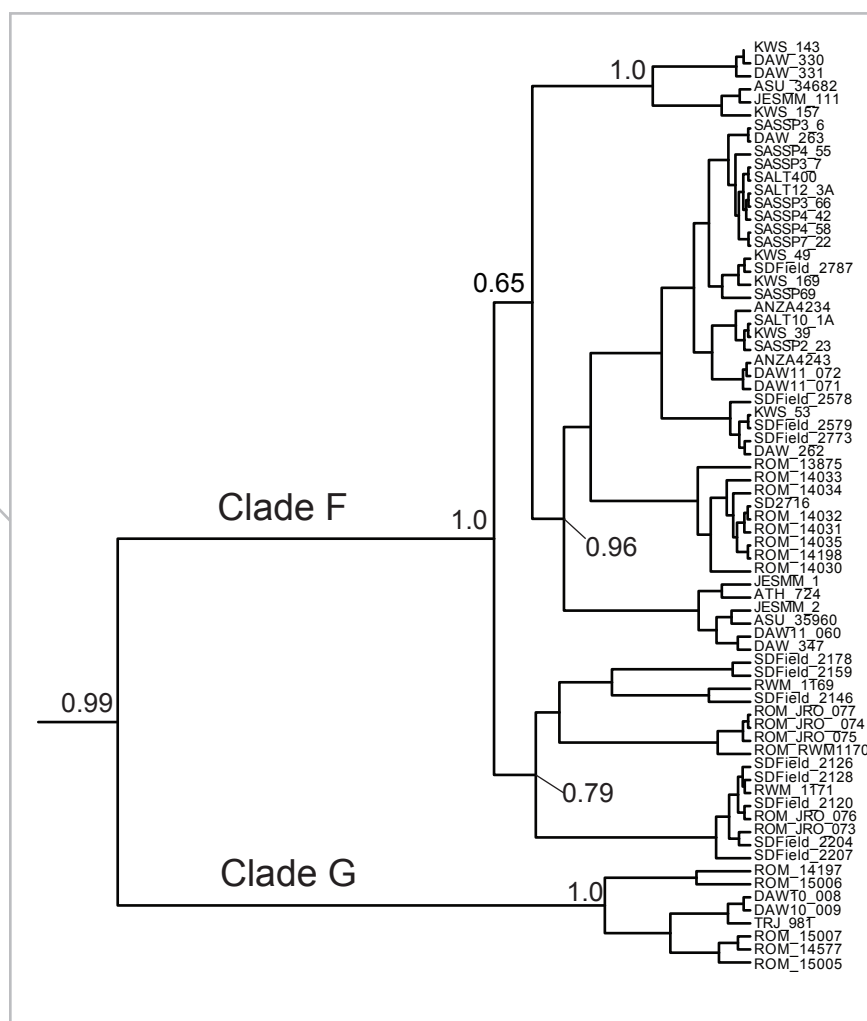

Supplement: Figure S3 — Colorado lineage pruned from the full mtDNA phylogeny, shown in the upper left. Numbers at nodes represent posterior probability support values for individual clades. (PDF) [file pone.0097494.s003.pdf]

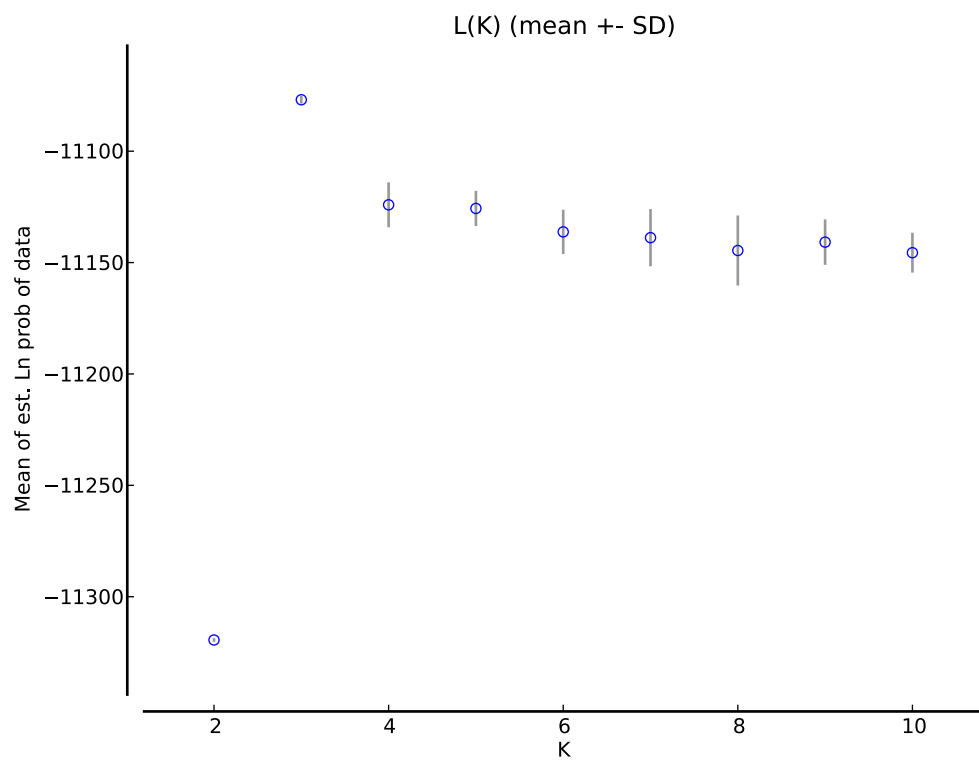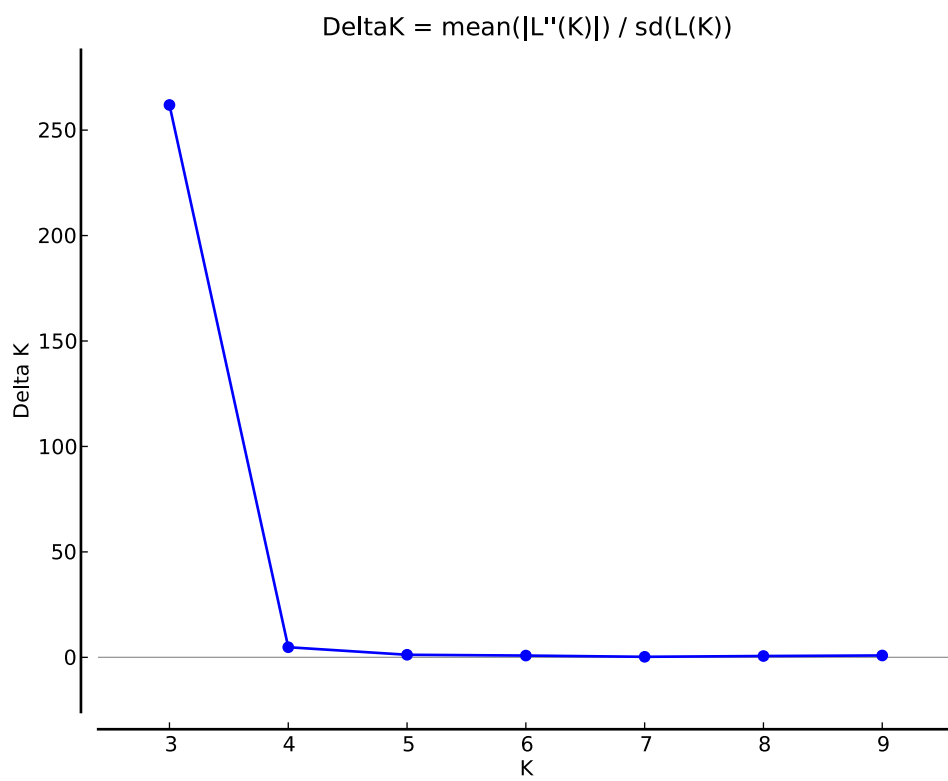

Supplement: Figure S4 — Results from the mean lnP(D| K ) score against the Kmax and the Δ K criterion of Evanno et al. [72] . (PDF) [file pone.0097494.s004.pdf]

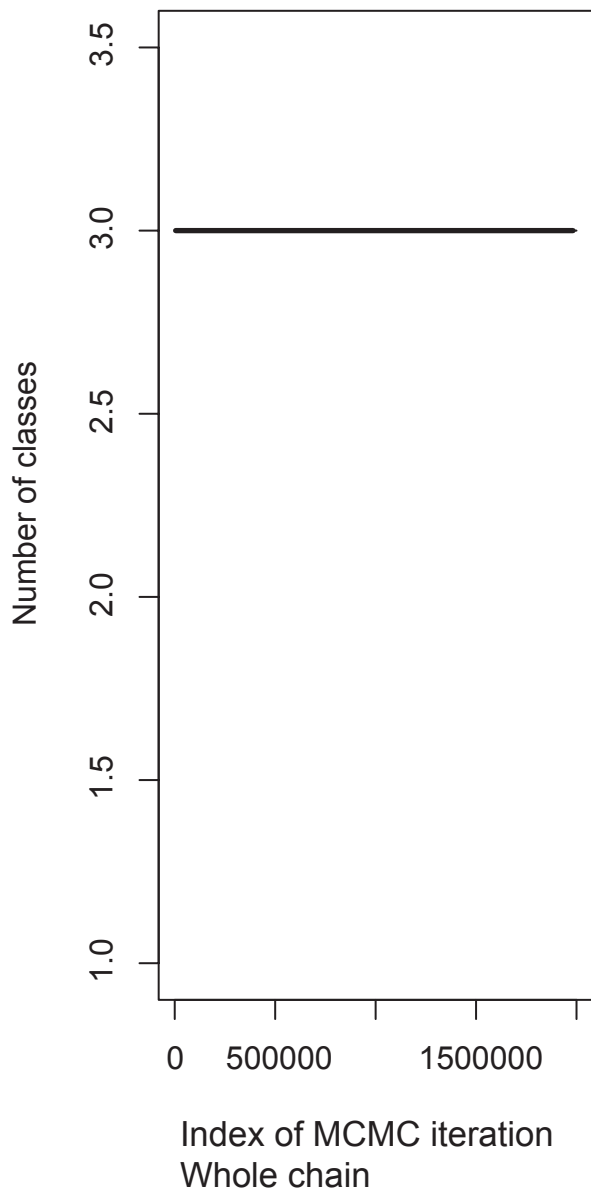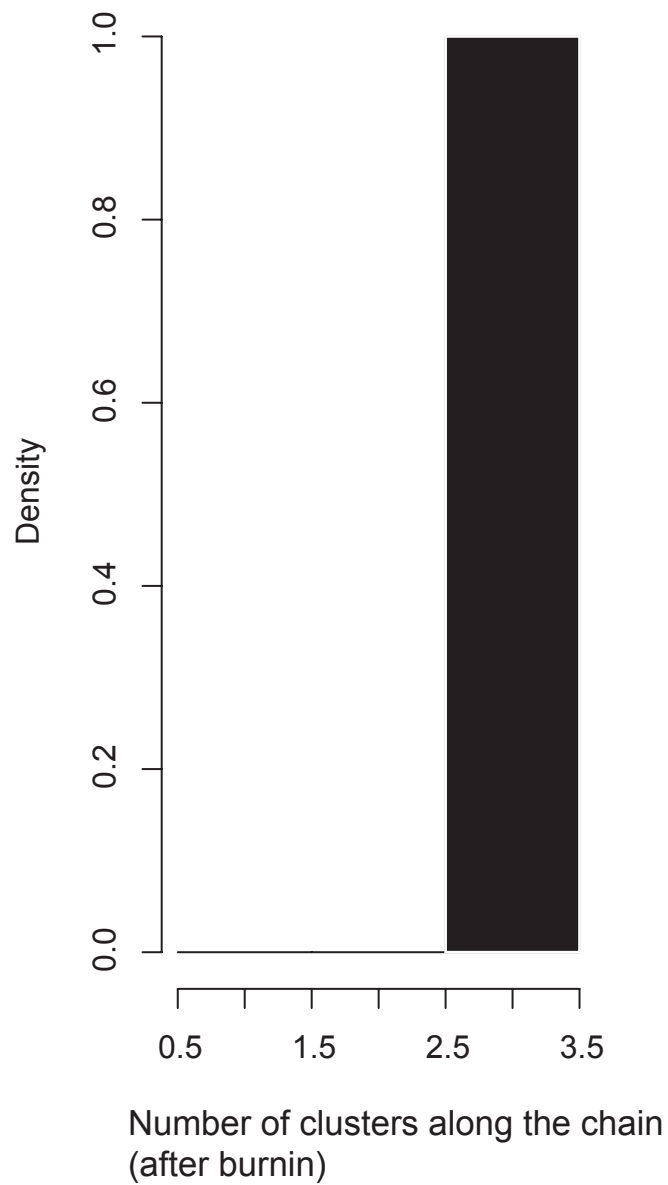

Supplement: Figure S5 — Posterior density of the number of clusters ( K ) from the MCMC analysis of genetic structure across Arizona using geneland. (PDF) [file pone.0097494.s005.pdf]
