# Supplementary material for: Fuzzy Boundaries: Color and Gene Flow Patterns among Parapatric Lineages of the Western Shovel-Nosed Snake and Taxonomic Implication
Source: PLoS One. 2014 May 21;9(5):e97494. doi: 10.1371/journal.pone.0097494 (PMC4029750; doi:10.1371/journal.pone.0097494)
Supplement: Figure S2 — Sonoran lineage pruned from the full mtDNA phylogeny, shown in the upper left. Numbers at nodes represent posterior probability support values for individual clades. (PDF) [file pone.0097494.s002.pdf]

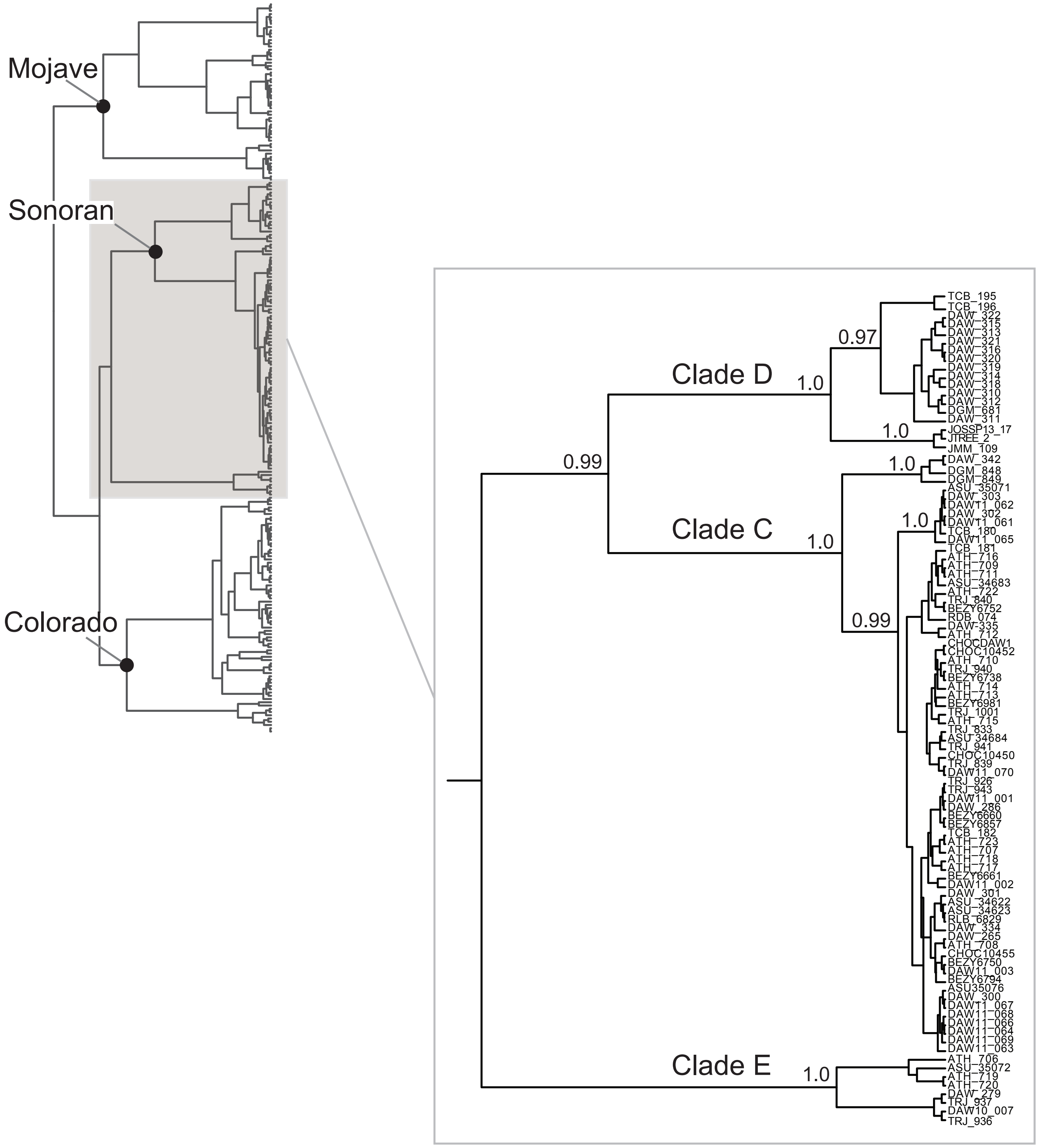
Mojave
Sonoran
Colorado
Clade D
Clade C
Clade E
0.99
0.97
1.0
1.0
1.0
1.0
1.0
0.99
1.0
TCB_195
TCB_196
DAW_322
DAW_315
DAW_313
DAW_321
DAW_316
DAW_320
DAW_319
DAW_314
DAW_318
DAW_310
DAW_312
DGM_681
DAW_311
JOSSP13_17
JTREE_2
JMM_109
DAW_342
DGM_848
DGM_849
ASU_35071
DAW_303
DAW11_062
DAW_302
DAW11_061
TCB_180
DAW11_065
TCB_181
ATH_716
ATH_709
ATH_711
ASU_34683
ATH_722
TRJ_840
BEZY6752
RDB_074
DAW_335
ATH_712
CHOCDAW1
CHOC10452
ATH_710
TRJ_940
BEZY6738
ATH_714
ATH_713
BEZY6981
TRJ_1001
ATH_715
TRJ_833
ASU_34684
TRJ_941
CHOC10450
TRJ_839
DAW11_070
TRJ_926
TRJ_943
DAW11_001
DAW_286
BEZY6660
BEZY6857
TCB_182
ATH_723
ATH_707
ATH_718
ATH_717
BEZY6661
DAW11_002
DAW_301
ASU_34622
ASU_34623
RLB_6829
DAW_334
DAW_265
ATH_708
CHOC10455
BEZY6750
DAW11_003
BEZY6794
ASU35076
DAW_300
DAW11_067
DAW11_068
DAW11_066
DAW11_064
DAW11_069
DAW11_063
ATH_706
ASU_35072
ATH_719
ATH_720
DAW_279
TRJ_937
DAW10_007
TRJ_936
